# Supplementary material for: A Critical View of the Application of the APEX Software (Aqueous Photochemistry of Environmentally-Occurring Xenobiotics) to Predict Photoreaction Kinetics in Surface Freshwaters
Source: Molecules. 2019 Dec 18;25(1):9. doi: 10.3390/molecules25010009 (PMC7017383; doi:10.3390/molecules25010009)
Supplement: Supplementary file 1 [file molecules-25-00009-s001.zip › 3.2.4_gcc-4.4.0.zip!/3.2.4_gcc-4.4.0/3.2.4_gcc-4.4.0/doc/octave/html/interpreter/Basic-Usage-of-Cell-Arrays.html]

Basic Usage of Cell Arrays - Untitled


Next: Creating Cell Arrays,
Up: Cell Arrays


---

#### 6.2.1 Basic Usage of Cell Arrays

As an example, the following code creates a cell array containing a
string and a 2-by-2 random matrix

```
     c = {"a string", rand(2, 2)};
```

To access the elements of a cell array, it can be indexed with the {
and } operators. Thus, the variable created in the previous example
can be indexed like this:

```
     c{1}
          => ans = a string
```

As with numerical arrays several elements of a cell array can be
extracted by indexing with a vector of indexes

```
     c{1:2}
          => ans =
     
               (,
                 [1] = a string
                 [2] =
     
                    0.593993   0.627732
                    0.377037   0.033643
     
               ,)
```

The indexing operators can also be used to insert or overwrite elements
of a cell array. The following code inserts the scalar 3 on the
third place of the previously created cell array

```
     c{3} = 3
          => c =
     
              {
                [1,1] = a string
                [1,2] =
     
                   0.593993   0.627732
                   0.377037   0.033643
     
                [1,3] =  3
              }
```

Details on indexing cell arrays are explained in Indexing Cell Arrays.

In general nested cell arrays are displayed hierarchically as in the
previous example. In some circumstances it makes sense to reference
them by their index, and this can be performed by the `celldisp`
function.

— Function File: **celldisp** (c, name)  
> Recursively display the contents of a cell array. By default the values
> are displayed with the name of the variable c. However, this name
> can be replaced with the variable name.
>
>
>
> **See also:** disp.

To test if an object is a cell array, use the `iscell`
function. For example:

```
     iscell(c)
          => ans = 1
     
     iscell(3)
          => ans = 0
```

— Built-in Function: **iscell** (x)  
> Return true if x is a cell array object. Otherwise, return
> false.
